# Cognitive Facilitation and Antioxidant Effects of an Essential Oil Mix on Scopolamine-Induced Amnesia in Rats: Molecular Modeling of In Vitro and In Vivo Approaches

**DOI:** 10.3390/molecules25071519

**Published:** 2020-03-27

**Authors:** Razvan Stefan Boiangiu, Ion Brinza, Monica Hancianu, Ilkay Erdogan Orhan, Gokcen Eren, Elife Gündüz, Halis Ertas, Lucian Hritcu, Oana Cioanca

**Affiliations:** 1 Department of Biology, Faculty of Biology, Alexandru Ioan Cuza University of Iasi, 700506 Iasi, Romania; boiangiu.razvan@yahoo.com (R.S.B.); ionbrinza995@gmail.com (I.B.); 2 Department of Pharmacognosy, Faculty of Pharmacy, Grigore T. Popa University of Medicine and Pharmacy Iasi, 700115 Iasi, Romania; mhancianu@yahoo.com (M.H.); oana.cioanca@gmail.com (O.C.); 3 Department of Pharmacognosy, Faculty of Pharmacy, Gazi University, 06330 Ankara, Turkey; iorhan@gazi.edu.tr; 4 Department of Pharmaceutical Chemistry, Faculty of Pharmacy, Gazi University, 06330 Ankara, Turkey; gokcene@gazi.edu.tr; 5 Talya Herbal Company, R & D Laboratory, Kepez, 07190 Antalya, Turkey; arge@talyabitkisel.com (E.G.); halis@talyabitkisel.com (H.E.)

**Keywords:** essential oil mix, scopolamine, memory, oxidative stress, molecular docking simulation

## Abstract

The present study investigated the capability of an essential oil mix (MO: 1% and 3%) in ameliorating amnesia and brain oxidative stress in a rat model of scopolamine (Sco) and tried to explore the underlying mechanism. The MO was administered by inhalation to rats once daily for 21 days, while Sco (0.7 mg/kg) treatment was delivered 30 min before behavioral tests. Donepezil (DP: 5 mg/kg) was used as a positive reference drug. The cognitive-enhancing effects of the MO in the Sco rat model were assessed in the Y-maze, radial arm maze (RAM), and novel object recognition (NOR) tests. As identified by gas chromatography–mass spectrometry (GC–MS), the chemical composition of the MO is comprised by limonene (91.11%), followed by γ-terpinene (2.02%), β-myrcene (1.92%), β-pinene (1.76%), α-pinene (1.01%), sabinene (0.67%), linalool (0.55%), cymene (0.53%), and valencene (0.43%). Molecular interactions of limonene as the major compound in MO with the active site of butyrylcholinesterase (BChE) was explored via molecular docking experiments, and Van der Waals (vdW) contacts were observed between limonene and the active site residues SER198, HIS438, LEU286, VAL288, and PHE329. The brain oxidative status and acetylcholinesterase (AChE) and BChE inhibitory activities were also determined. MO reversed Sco-induced memory deficits and brain oxidative stress, along with cholinesterase inhibitory effects, which is an important mechanism in the anti-amnesia effect. Our present findings suggest that MO ameliorated memory impairment induced by Sco via restoration of the cholinergic system activity and brain antioxidant status.

## 1. Introduction

Neurodegeneration can be defined as forfeiture of neuron structure and function in a progressive way, which is associated with various diseases such as Alzheimer’s disease (AD), Parkinson’s disease, Huntington disease, amyotrophic lateral sclerosis (ALS), multiple sclerosis (MS), etc. [1]. Among them, AD is estimated to affect approximately 40 million people all over the world, with an increasing incidence tendency [2]. Since AD has multifactorial and complex pathophysiology, a pair of hypotheses have been so far proposed, known as the ”amyloid hypothesis” and “cholinergic hypothesis.” Based on the cholinergic hypothesis approach, the most prescribed drug class against the disease is AChE inhibitors, as an apparent deficit in acetylcholine (ACh) is shown to occur in the brains of AD patients. Therefore, inhibition of AChE is suggested to be a promising strategy [3], which restores the memory deficits in AD patients. Despite repeated failures in the formulation of preventive and disease-modifying therapies, the AChE inhibitors appear to play a crucial role in controlling the symptoms and potentially slowing the rate of AD progression [4]. Notably, research also suggested that people with mild cognitive impairment (MCI) and dementia exhibited an increased risk of developing anxiety and depression as compared to people with normal cognitive function [5]. Also, it has been demonstrated that oxidative stress is a pivotal factor to the progression of AD through three pathways: macromolecule peroxidation, amyloid-beta (Aβ) metal ion redox potential, and mitochondrial dysfunction, all of which affect cell homeostasis, the generation of reactive oxygen species (ROS), and the up-regulation of Aβ and *p*-tau formation [6].

Essential oils are a mixture of extremely complex, volatile, naturally derived compounds that are derived from plants as secondary metabolites. They are found to be abundant in leaves, seeds, flowers, barks, and rhizomes and are commonly produced by cold pressing and hydro-distillation methods [7]. Their major constituents include monoterpene hydrocarbons, sesquiterpene hydrocarbons, oxygenated sesquiterpenes, oxygenated monoterpenes, and esters [8]. A short review of in vivo studies of 13 essential oils together with clinical trial data of *Salvia officinalis* L., *S. lavandulifolia* Vahl., *Melissa officinalis* L., *Lavandula angustifolia* Mill., and *Rosmarinus officinalis* L. revealed their antioxidant and anti-AChE potential, as well as anti-amyloid and memory-enhancing activities [9]. De Araujo et al. [10] point out the antioxidant and antimicrobial activities of *Citrus aurantium var. dulcis* Hayne essential oil. Falls et al. [11] reported the amelioration of neurodegeneration and cognitive impairment in an experimental model of stressed mice following exposure to *Citrus lemon* (L.) Osbeck essential oil for three weeks. Oskouie et al. [12] demonstrated the beneficial therapeutic effect of *Lavandula angustifolia* using an Aβ1-42 rat model of AD through the investigation of serum metabolic features using NMR metabolomics. Kennedy and Scholey [13] reported the psychopharmacology of European herbs with cognition-enhancing properties. Altogether, including *R. officinalis* L., these species may well provide effective and well-tolerated treatments for dementia, either alone, in combination, or as an adjunct to conventional treatments. Hasanein et al. [14] demonstrated the preventive effects of *S. officinalis* L. against learning and memory deficit induced by diabetes in rats by possible hypoglycemic and antioxidant mechanisms. Therefore, the present study was undertaken to highlight the bioactive components of the MO and to evaluate the possible effects on memory performance and brain antioxidant status in a Sco-induced rat model of amnesia.

## 2. Results and Discussion

### 2.1. The Chemical Composition of the Essential Oil Mix

The MO formulated for motivation and enhancing mood was analyzed by gas chromatography–mass spectrometry (GC–MS). The data showed that limonene was the major compound (91.11%), followed by other minor constituents, γ-terpinene (2.02%), β-myrcene (1.92%), β-pinene (1.76%), α-pinene (1.01%), sabinene (0.67%), linalool (0.55%), cymene (0.53%), and valencene (0.43%), which constituted 100% of the oil mix. The MO tested was expected to contain limonene as the leading component as limonene is well-known to be the major monoterpene in *Citrus* essential oils, which was confirmed by our analysis. Mounting evidence suggested that limonene exhibited anti-dementia effects against Sco through inhibition of AChE activity in rats [15]. Further, among the components evidenced in the essential oil from alpine needle leaves of *Abies koreana*, γ-terpinene exhibited a memory-enhancing effect on Sco-induced amnesia in mice [16]. Lee et al. [17] also reported that α-pinene has an ameliorative effect on Sco-induced learning and memory impairment in C57BL/6 mice. These findings indicated that the memory-ameliorating effect of the MO and anti-AChE properties could be attributed to the presence of these compounds.

### 2.2. Molecular Docking Simulations

**Figure 1:**
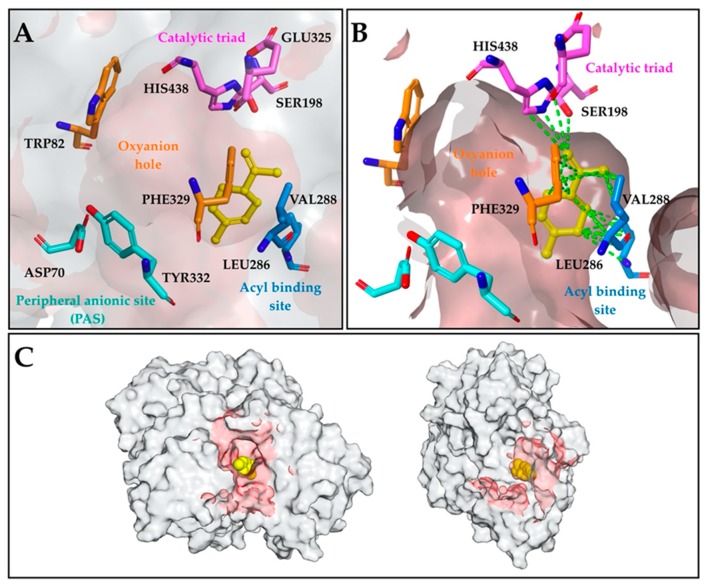
Proposed binding mode of limonene in the binding site of hBChE (PDB: 4TPK). (**A**) Active site residues are represented as a stick, and limonene is presented as the yellow ball and stick mode; (**B**) the vdW contacts are represented as green dotted lines; (**C**) the view of the complex showing the surface of hBChE (white) and limonene (yellow).

In order to analyze the molecular interactions of limonene and BChE at an atomic level, molecular docking studies on hBChE (PDB: 4TPK) was performed using the Glide module implemented in Schrödinger Small-Molecule Drug Discovery Suite. As depicted in Figure 1, limonene was located deeper into the binding pocket and occupied the region between the catalytic triad and the acyl binding site. Limonene exhibited binding energy of −6.522 kcal/mol against BChE, which was modulated by vdW contacts with the catalytic triad member residues SER198 and HIS438, the acyl binding site comprising residues LEU286 and VAL288, and the oxyanion hole residue PHE329. It was suggested that these hydrophobic interactions contributed to the stabilization of limonene; the BChE complex prevented substrate access to the catalytic triad, which is found to be correlated with the inhibition% value of 69.12 of the MO containing mainly limonene.

### 2.3. Cholinesterase Inhibitory Effect of the Essential Oil Mix

The MO formulation was tested against AChE and BChE using microtiter enzyme inhibition assays at 200 µg/mL. It displayed 24.97 ± 5.57% and 69.12 ± 4.78% of inhibition against AChE and BChE, respectively, where galantamine as the reference drug showed 96.02 ± 1.98% and 82.35 ± 5.36% against AChE and BChE, respectively. The IC_50_ value of the MO for BChE was calculated as 1.096 ± 0.043 µg/mL. The results of our study are in line with other studies reported in vitro AChE inhibitory activity of various essential oils. This may likely contribute to the presence of different bioactive compounds. Mukherjee et al. [18] demonstrated in vitro AChE inhibitory activity of the essential oil from *Acorus calamus* and its constituents. The IC_50_ values were obtained for the hydroalcoholic extract, the essential oil, beta-asarone, and alpha-asarone and were 182.31 ± 16.78 μg/mL, 10.67 ± 0.81 μg/mL, 3.33 ± 0.02 μM, and 46.38 ± 2.69 μM, respectively. Souza et al. [19] reported the AChE inhibitory activity of the essentials oils from *Myrceugenia myrcioides* (Cambess.) O. Berg and *Eugenia riedeliana* O. Berg. with an IC_50_ value of 67.3 μg/mL. Dohi et al. [20] showed the AChE inhibitory activity of commercially available essential oils extracted from *Artemisia dracunculus* L., *Inula graveolens* L., *Lavandula officinalis* Chaix, and *Ocimum sanctum* L. The authors reported that all of the oils showed potent inhibitory activity (IC_50_ = 0.058 mg/mL). Consistent with these results, our MO exhibited an intense AChE inhibitory activity, which could support the improvement of memory functions as assessed in behavioral approaches.

### 2.4. The Effects of the Essential Oil Mix on Cognitive Functions in Behavioral Tasks

Based on the findings of the in vitro experiments, we continued in vivo investigations of the MO in Sco–induced amnesic AD-like rat. Clinically, AD has been characterized by behavioral and cognitive impairments, in which patients appear to lose their memory slowly and cannot maintain their physiological functions [21]. Sco, a competitive muscarinic ACh receptor antagonist, has been reported to actively induce cognitive deficits in rats following other molecular events [22]. Thus, we used Sco (0.7 mg/kg) to produce AD-like memory deficits in rats, and DP (5 mg/kg), an approved AChE inhibitor, as a positive reference drug.

**Figure 2:**
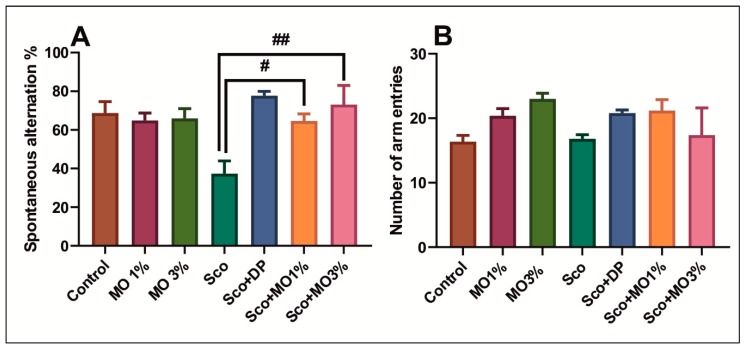
The essential oil mix (MO: 1% and 3%) improved memory performance in the Y-maze test. (**A**) Spontaneous alternation percentage in different groups. (**B**) The number of arm entries (locomotor activity) in different groups. Values are means ± SEM (*n* = 5). For Tukey’s post hoc analyses: (**A**) Sco vs. Sco + MO1%: # *p* < 0.01, and Sco vs. Sco + MO3%: ## *p* < 0.001.

Figure 2 shows the effects of Sco (0.7 mg/kg) and MO (1% and 3%) treatment on spontaneous alternation behavior and locomotor activity (number of arm entries) in the Y-maze test. Sco (0.7 mg/kg) injection significantly decreased short-term memory performance, as evidenced by decreased spontaneous alternation percentage compared with the control group (*p* < 0.01). Both doses of MO, but especially the higher one (3%), significantly improved the spontaneous alternation percentage (*p* < 0.001) decreased by Sco, as compared to Sco alone-treated rats. DP (5 mg/kg), a positive reference drug, and both doses of MO (1% and 3%) significantly increased spontaneous alternation percentage close to the control group, suggesting positive effects on short-term memory (Figure 2A). Nevertheless, the improvement of the short-term memory could not be attributed to locomotor activity, as revealed by the number of arm entries, because no significant differences between groups were noticed (Figure 2B). These results suggest that MO produced an improving effect on short-term memory.

**Figure 3:**
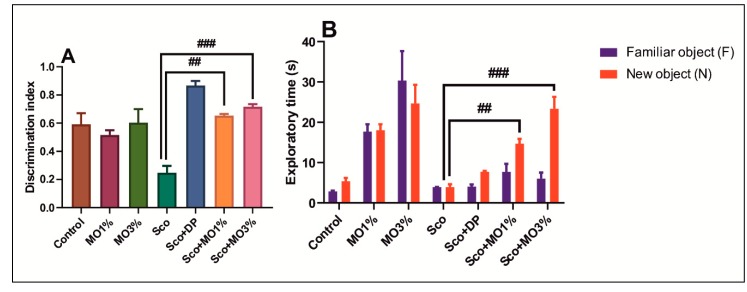
The essential oil mix (MO: 1% and 3%) improved the consolidation of NOR memory. (**A**) Discrimination index in different groups. (**B**) Exploratory time (s) in different groups. Values are means ± SEM (*n* = 5). For Tukey’s post hoc analyses: (**A**) Sco vs. Sco + MO1%: ## *p* < 0.001, and Sco vs. Sco + MO3%: ### *p* < 0.0001; (**B**) Sco vs. Sco + MO1%: ## *p* < 0.001, and Sco vs. Sco + MO3%: ### *p* < 0.0001.

Figure 3 reports the effects of Sco (0.7 mg/kg) and MO (1% and 3%) treatments on the consolidation of NOR memory. Sco (0.7 mg/kg) injection significantly decreased the DI value as compared with the control group (*p* < 0.001). Both doses of MO, but especially the higher one (3%), were found to be effective in increasing the DI value as compared to Sco alone-treated rats. DP (5 mg/kg), a positive reference drug, and both doses of MO (1% and 3%) significantly increased the DI value, suggesting positive effects on the consolidation of NOR memory (Figure 3A). Moreover, Sco treatment decreased the TN as compared to the control group (*p* < 0.01). Inhalation of the MO (1% and 3%), but especially the dose of 3%, significantly increased the TN rather than TF as compared to Sco alone-treated rats. (Figure 3B). In the NOR test, DP (5 mg/kg), a positive reference drug, also increased TN. These results suggest that MO produced an improving effect on the consolidation of NOR memory.

**Figure 4:**
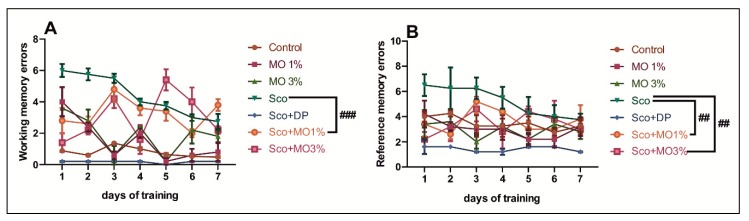
The essential oil mix (MO: 1% and 3%) improved spatial memory in the radial arm maze test. (**A**) Working memory errors in different groups. (**B**) Reference memory errors in different groups. Values are means ± SEM (*n* = 5). For Tukey’s post hoc analyses: (**A**) Sco vs. Sco + MO1%: ### *p* < 0.0001; (**B**) Sco vs. Sco + MO1%: ## *p* < 0.001, and Sco vs. Sco + MO3%: ## *p* < 0.001.

Figure 4 shows the effects of Sco (0.7 mg/kg) and MO (1% and 3%) treatment on working memory and reference memory in the RAM. Sco (0.7 mg/kg) injection significantly decreased working memory performance, as evidenced by increased working memory errors compared with the control group (*p* < 0.0001) (Figure 4A). Consequently, a significant decrease of reference memory performance, as evidenced by increased reference memory errors, was observed, following Sco (0.7 mg/kg) administration as compared to the control group (*p* < 0.001) (Figure 4B). Both doses of MO and DP (5 mg/kg), a positive reference drug, significantly ameliorated the working memory errors (*p* < 0.0001 for MO1%) (Figure 4A), and the reference memory errors (*p* < 0.001 for MO1% and *p* < 0.001 for MO3%) (Figure 4B) were decreased by Sco, as compared to Sco alone-treated rats. These results suggest that MO produced an improving effect on working memory and reference memory.

Our results are in perfect agreement with those obtained by other groups, and demonstrated that the MO exhibited a cognitive facilitating profile, which could be attributed to the synergistic effects of the components of the essential oil such as *C. aurantium dulcis* oil, *C. lemon* oil, *L. angustifolia* oil, *R. officinalis* oil, and *S. officinalis* oil. Lee et al. [23] demonstrated the ameliorating effect of *C. aurantium* extract and nobiletin on Aβ1-42-induced memory impairment in mice by regulating anti-apoptotic mechanisms, including reduced AChE activity in the cortex and hippocampus of the cognitive deficit mouse model. Falls et al. [11] reported the amelioration of neurodegeneration and cognitive impairment by *Citrus lemon* essential oil in an experimental model of stressed mice, by the reduction in AChE activity and by elevated GSH, SOD, and CAT through the decrease in raised plasma corticosterone levels. Xu et al. [24] showed the protective effect of *L. angustifolia* Mill. essential oil on Sco-induced cognitive deficits in mice and H_2_O_2_-induced cytotoxicity in PC12 cells via modulating oxidative stress and AChE activity. Also, our group already reported that two different lavender essential oils from *L. angustifolia* ssp. *angustifolia* Mill. and *L. hybrida* Rev. reversed spatial memory deficits induced by Sco. Ozarowski et al. [25] reported an improvement of long-term memory impairment in rats following the administration of *R. officinalis* L. leaf extract via inhibition of the rat brain AChE activity. Our group recently demonstrated that *R. officinalis* essential oil improved Sco-induced neurobehavioral changes via restoration of cholinergic function and brain antioxidant status in zebrafish (*Danio rerio*). Finally, various reports demonstrated that *Salvia officinalis* L. exhibited cognitive-enhancing potential and protective effects, which can be explained by its cholinesterase inhibiting properties and antioxidant mechanisms [26,27,28]. Consistent with these results, our findings suggested the ability of our MO to beneficially modulate and improve the cholinergic neuronal transmission and cognitive performance within dementia-related conditions.

### 2.5. Effects of the Essential Oil Mix on Brain AChE in Sco-Treated Rats

**Figure 5:**
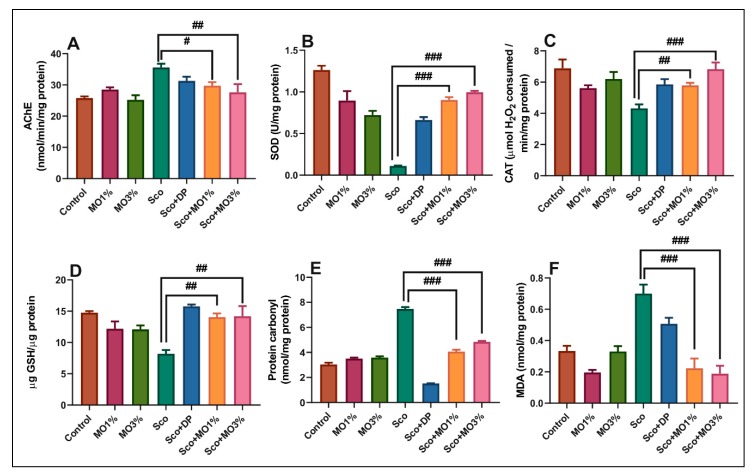
The essential oil mix (MO: 1% and 3%) exhibited an anti-AChE effect and improved brain antioxidant status. The enzyme’s specific activities: (**A**) AChE, (**B**) SOD, (**C**) CAT, and (**D**) GSH, (**E**) protein carbonyl, and (**F**) MDA level. Values are means ± SEM. (*n* = 5). For Tukey’s post hoc analyses: (**A**) Sco vs. MO1%: # *p* < 0.01 and Sco vs. MO3%: ## *p* < 0.001; (**B**) Sco vs. Sco + MO1%: ### *p* < 0.0001, and Sco vs. Sco + MO3%: ### *p* < 0.0001; (**C**) Sco vs. Sco + MO1%: ## *p* < 0.001, and Sco vs. Sco + MO3%: ### *p* < 0.0001; (**D**) Sco vs. Sco + MO1%: ## *p* < 0.001, and Sco vs. Sco + MO3%: ## *p* < 0.001; (**E**) Sco vs. Sco + MO1%: ### *p* < 0.0001, and Sco vs. Sco + MO3%: ### *p* < 0.0001 and (**F**) Sco vs. Sco + MO1%: ### *p* < 0.0001, and Sco vs. Sco + MO3%: ### *p* < 0.0001.

According to the cholinergic hypothesis, a low level of ACh is the leading cause of the cognitive decline in AD patients. Accumulation of data suggests that the AChE plays an essential role in the regulation of several physiological reactions by hydrolyzing the neurotransmitter ACh in cholinergic synapses [29]. Therefore, the inhibition of AChE may be a better therapeutic strategy in the treatment of AD. Sco increased brain AChE activity in rats as compared to the control group (*p* < 0.001) (Figure 5A). Both doses of MO (1% and 3%), but especially the dose of 3%, decreased brain AChE activity that was increased by Sco (*p* < 0.01 for MO1% and *p* < 0.001 for MO3%). As expected, DP (5 mg/kg), an AChE inhibitor, significantly prevented the increased AChE activity induced by Sco. Moreover, our results suggested that the MO has the potential to ameliorate cognitive dysfunction in Sco-induced AD-like rats, likely by inhibiting AChE activity.

### 2.6. Effects of the Essential Oil Mix on Brain Oxidative Status in Sco-Treated Rats

Accumulated data suggested that the oxidative stress produced by ROS may play a key role in in the development of AD in the aging population [30]. The brain usually needs higher levels of oxygen to perform its extensive synaptic functions and is highly susceptible to oxidative stress, especially in the hippocampal and cortex regions [31]. The excessive synthesis of ROS allows thiol-and lipid-dependent cell membrane peroxidation to inflict a neurotoxic mechanism, reducing hippocampal plasticity, thereby having direct involvement in the pathogenesis of AD [32]. Besides the behavioral and cognitive regression, Sco-induced AD rats often clearly mimic the oxidative stress event of AD development, although its exact oxidative damage mechanism is uncertain [33]. The underlying mechanism for enhancing memory found in specific behavioral tests have been further explored by examining biochemical parameters such as SOD, CAT, GSH, and MDA.

Sco decreased SOD (*p* < 0.0001) (Figure 5B) and CAT (*p* < 0.001) (Figure 5C) specific activities and then reduced the total content of reduced GSH (*p* < 0.001) (Figure 5D) and increased protein carbonyl (*p* < 0.0001) (Figure 5E) and MDA (*p* < 0.0001) (Figure 5F) levels as compared to the control group. Both doses of MO (1% and 3%), but especially 3%, increased brain SOD and CAT specific activities, which were decreased by Sco. Furthermore, MO increased brain content of reduced GSH along with reduced levels of protein carbonyl and MDA in the rat brain.

The results suggested that MO exhibits neuroprotective effects against oxidative stress. Haider et al. [34] reported that naringenin, a flavonoid from *C. aurantium*, protects AlCl_3_/D-galactose-induced neurotoxicity in a rat model of AD via attenuation of AChE levels and inhibition of oxidative stress. Nobiletin, from *C. lemon* peel, was found to be a potent candidate for the prevention of AD through the inhibition of oxidative stress, apoptosis, and inflammation via suppression of interleukin-1β, tumor necrosis factor-α, nitric oxide (NO), and prostaglandin E2 production in response to Aβ_25-35_ stimulation [35]. Supporting evidence suggested that lavender oil (*Lavandula stoechas* L.) is a very potent antioxidant attenuating dementia in Sco-induced memory deficit in mice brains. The authors also demonstrated that the methanolic extract of *L. stoechas* reduced brain AChE and MDA levels while improved SOD, CAT, and GSH levels [36]. Additionally, our group reported that the lavender essential oils from *L. angustifolia* ssp. *angustifolia* Mill. and *L. hybrida* Rev. increased brain antioxidant enzyme activities (SOD, GPX, and CAT), the total content of reduced GSH, and reduced lipid peroxidation (MDA level) in rat temporal lobe homogenates of the Sco-treated animals, suggesting antioxidant potential. Also, DNA cleavage patterns were absent in the lavender groups, suggesting antiapoptotic activity. Rosmarinic acid, an antioxidant agent from *R. officinalis,* was found to ameliorate H_2_O_2_-induced oxidative stress in L02 cells through mitogen-activated protein kinases (MAPK) and nuclear factor E2-related factor 2 (Nrf2) pathways [37].

Moreover, recently our group reported that the administration of *R. officinalis* to zebrafish abolished AChE alteration and brain oxidative stress induced by Sco [38]. *S. officinalis* has been observed to have anti-inflammatory and antioxidant effects on lipopolysaccharide (LPS)-induced inflammation in rats [39] and attenuated bleomycin-induced oxidative stress and lung fibrosis in rats [40]. In line with early results, we assume that the presence of the bioactive compounds within the MO may potentially lead to its increased antioxidant potential.

**Figure 6:**
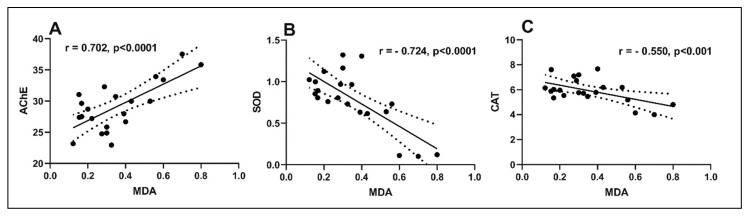
Correlation analyses between biochemical parameters and lipid peroxidation (Pearson’s correlation, *n* = 5): (**A**) AChE vs. MDA (*r* = 0.702, *p* < 0.0001); (**B**) SOD vs. MDA (*r* = −0.724, *p* < 0.0001) and (**C**) CAT vs. MDA (*r* = −0.550, *p* < 0.001). Data expressed are AChE (nmol/min/mg protein), SOD (U/mg protein), CAT (μmol H2O2 consumed/min/mg protein), and MDA (nmol/mg protein).

In the present study, the Pearson correlation coefficient (*r*) was used to test the linear association between antioxidant enzymes and lipid peroxidation (Figure 6). A high positive correlation between the AChE vs. MDA (*n* = 5, *r* = 0.702, *p* < 0.0001) (Figure 6A) was observed. The positive value of the *r* indicates that the decreased AChE activity is well correlated with a low level of MDA, a marker of lipid peroxidation. Similarly, strong negative correlations were evidenced by linear regression between SOD vs. MDA (*n* = 5, *r* = −0.724, *p* < 0.001) (Figure 6B) and CAT vs. MDA (*n* = 5, *r* = −0.550, *p* < 0.001) (Figure 6C). In this case, the negative values of the *r* indicate that increasing antioxidant enzyme activity is well-correlated with decreasing of MDA level, supporting the neuroprotective profile of MO. No significant correlation between behavioral scores and biochemical parameters was observed. Wang et al. [41] demonstrated a relation by correlation analysis among antioxidant capacity, anticancer ability, and flavonoid composition of 35 citrus (*C. reticulata* Blanco) varieties. Xu et al. [42] demonstrated a relationship between the protective effect of lavender essential oil and its main component linalool against the cognitive deficits induced by d-galactose and AlCl_3_ in mice. Habtemariam et al. [43] revealed a relation between the diterpenes from *R. officinalis* and its antioxidant potential, anti-inflammatory mechanisms, and Aβ aggregation, and anti-AChE activity with relevance to the AD pathology. Finally, Ghorbani et al. [44] disclosed a correlation between the pharmacological properties of *S. officinalis* such as anticancer, anti-inflammatory, antinociceptive, antioxidant, antimicrobial, antimutagenic, antidementia, hypoglycemic, and hypolipidemic effects and its chemical constituents. Thus, in the present study, our MO supported memory formation and exhibited an antioxidant profile that could be perfectly attributed to the inhibition of AChE that parallels with the restoration of antioxidant enzyme activities and decreasing of lipid peroxidation levels.

## 3. Materials and Methods

### 3.1. Essential Oil and Plant Materials

The MO tested was formulated based on a mixture of the essential oils from five aromatic plants, including sweet orange (68%), lemon (30%), lavender (1%), sage (0.5%), and rosemary (0.5%). The corresponding plants, e.g., *Citrus aurantii dulcis*, *Citrus lemon*, *Lavandula angustifolia*, *Salvia officinalis*, and *Rosmarinus officinalis* collected throughout the Mediterranean region in Turkey between April–July 2019. The plants were identified the R&D laboratory of Talya Herbal Company (Antalya, Turkey), where the voucher specimens were also preserved.

### 3.2. Gas Chromatography-Mass Spectrometry (GC-MS) Analysis

Component analysis of the MO was performed using a gas chromatography- (Agilent 7890A) mass detector (Agilent 5975C) device by using a capillary column (HP Innowax Capillary; 60.0 m × 0.25 mm × 0.25 μm) at the Instrumental Analysis Laboratory of Western Mediterranean Research Institute (BATEM) in Antalya, Turkey. For analysis, the MO was diluted with *n*-hexane at the rate of 1:50. Helium with the flow rate of 0.8 mL/min was used as the carrier gas for the analysis. The sample was injected into the device at 1 μL with a split ratio of 1:40. Injector temperature was held at 25 °C, the column temperature program was set to be 10 min at 60 °C, 4 °C/min from 60 °C to 220 °C, 10 min at 220 °C. The total analysis time was 60 min following this temperature program. The scan range was 35–500 atomic mass units for the mass detector, and electron bombardment ionization was 70 eV. OIL ADAMS AND WILEY libraries data were used for the essential oil component identification. The amounts of the components were determined by using the FID detector, and identification of the components was determined by using the MS detector.

### 3.3. Molecular Docking Experiments

The molecular docking studies were carried out using the Glide docking protocol implemented in the Schrödinger Small-Molecule Drug Discovery Suite (Small-Molecule Drug Discovery Suite 2019-4, Schrödinger, LLC, New York, NY, USA, 2019). The compound, which was built via the builder panel in Maestro, was subjected to ligand preparation by LigPrep (Schrödinger Release 2019-4: LigPrep, Schrödinger, LLC, New York, NY, USA, 2019) using default conditions. The crystal structure of hBChE (PDB: 4TPK) [45] was retrieved from the Protein Data Bank. The protein was prepared using the Protein Preparation Wizard tool. Hydrogen atoms were added, followed by assignment of all atom charges and atom types. Finally, energy minimization and refinement of the structures were done up to 0.3 ÅRMSD by applying the OPLS3e force field. The centroid of the ligand (the coordinates: X: 4.02; Y: 10.77; Z: 13.86) was defined as the grid box with a size of 72 × 72 × 72 Å. vdW radius scaling factor 1.00, partial charge cutoff 0.25, and OPLS3e force filed were used for receptor grid generation. The compound prepared by LigPrep was docked into the proteins using the extra-precision (XP) docking mode of the Glide without using any constraints and a 0.80 vdW radius scaling factor and 0.15 partial charge cutoff with 3 runs and the post-docking minimization was performed with 10 poses per ligand [46]. The best conformation was chosen based on the lowest XP glide score.

### 3.4. Microtiter Enzyme Assays for AChE and BChE Inhibition

AChE and BChE inhibitory activity of the MO was determined by the slightly modified spectrophotometric method developed by Ellman et al. [47]. Briefly, 140 μL of sodium phosphate buffer (pH 8.0), 10 μL of DTNB, 20 μL of the test solution, and 20 μL of AChE/BChE solution were added by multichannel automatic pipette (Gilson´s Pipetman, Gilson, Inc., Paris, France) in a 96-well microplate and incubated for 10 min at 25 °C. The reaction was then initiated with the addition of 10 μL of acetylthiocholine iodide (ATC)/butyrylthiocholine chloride (BTC). Hydrolysis of ATC/BTC was monitored by the formation of the yellow 5-thio-2-nitrobenzoate anion as a result of the reaction of DTNB with thiocholines, catalyzed by enzymes at 412 nm utilizing a 96-well VersaMax Microplate Reader (Molecular Devices, San Jose, CA, USA). The measurements and calculations were evaluated by using Softmax PRO 4.3.2.LS software. The percentage of inhibition of AChE/BChE was determined by comparison of the reaction rates of samples vs. blank sample (ethanol in phosphate buffer, pH 8.0) using the formula I% = 100 – [(A1/A2) × 100], where A1 is the absorbance of the sample solutions at 412 nm and A2 is the average absorbance of the control solutions at the same wavelength. The results are given as mean ± standard deviation of the % inhibitions obtained from three experiments. Galantamine was used as the reference drug.

### 3.5. Animals

Wistar adult rats-males were purchased from Cantacuzino Institute (Bucharest, Romania) having an average weight of 350 g (±10g) at 4 months old. The animals were installed in a room under controlled conditions of light (a 12-h cycle starting at 08:00 h) and temperature (22 °C), having plenty of food and water ad libitum. All animal experiments were carried out according to the European Communities Council Directive (Directive 2010/63/EU) regarding the protection of animals used for scientific purposes with approval from the Ethical Committee (No. 15309/22.07.2019).

### 3.6. Drug Treatment and Group Division

**Figure 7:**
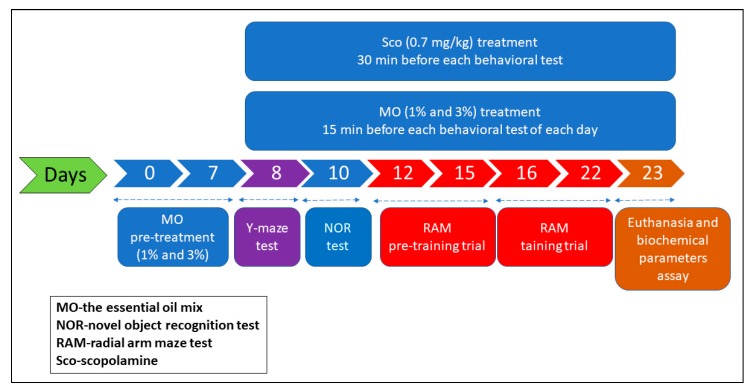
Schematic representation of drug treatment and behavioral analysis.

The scheme of drug treatments and behavioral measurements is summarized in Figure 7. For the behavior studies, rats were randomly divided into 7 groups (*n* = 5 animals/group) as follows: the control group, the MO treatment groups (MO1% and MO3%, Talya Herbal, Antalya, Turkey), the Sco (0.7 mg/kg b.w., i.p., Sigma-Aldrich, Darmstadt, Germany) group, the Sco received DP group (Sco + DP: as the positive control, 5 mg/kg b.w., i.p., Sigma-Aldrich, Darmstadt, Germany), and the Sco received by inhalation MO (Sco + MO1% and Sco + MO3%). Control, Sco + DP, and Sco groups received 1% Tween 80 solution through inhalation. MO was diluted with 1% Tween 80 (*v*/*v*). MO exposure (200 μL, either 1% or 3%) was via an electronic vaporizer (KBAYBO). Rats were pretreated by inhalation with MO (1% and 3%) for seven days before starting behavioral tests and continuously administered during behavior tests (21 days). DP (5 mg/kg, Sigma-Aldrich, Darmstadt, Germany) was dissolved in 0.9% physiological saline and injected i.p., once daily, 30 min before the Y-maze, NOR, and RAM tasks. Sco (0.7 mg/kg b.w., Sigma-Aldrich, Darmstadt, Germany) was intraperitoneally (i.p.) injected into rats 30 min before behavior tests [48].

### 3.7. Behavioral Analysis

#### 3.7.1. Y-maze

Spontaneous alternation behavior was evaluated in a single-session Y-maze as previously described by Jackson [49] and Postu et al. [50]. In the present study, the Y-maze used was built of Plexiglas having the following dimensions: 25 cm high, 35 cm long, 10 cm wide for each arm, and a central area in an equilateral triangular shape. At 15 min after inhaling MO (1% and 3%), each rat was positioned at the end of one arm and given the possibility to move for 8 min. Successive entry in each arm was considered as an alternation in behavior. A Logitech HD Webcam C922 Pro Stream camera (Logitech, Lausanne, Switzerland) recorded animal behavior, and the videos were analyzed using ANY-maze^®^ software (Stoelting CO, Wood Dale, IL, USA). The spontaneous alternation percentage was calculated as (number of alternation/total entries − 2) × 100. Between trials, the Y-maze was washed with a 10% ethanol solution.

#### 3.7.2. Novel Object Recognition

Novel object recognition (NOR) has been extensively used for determining alterations in cognitive functions as it can measure the preference of novelty in rodents. The preference of a novel object may indicate the existence of familiar object presentation in the memory of a rat [34]. Rats were trained and tested in a NOR test, as previously described by Foyet et al. [51]. The apparatus consisted of a black Plexiglas open field measuring 72 cm × 72 cm with 36 cm walls. In the ”sample” trial (T1) rats were individually placed into the field and were left to explore two identical objects positioned in two opposite corners of the apparatus. After T1, rats were moved to their home cages, and an inter-trial interval of 1 h was given. Subsequently, the ”choice” trial (T2) was performed. The rats explored the open field for 5 min in the presence of one familiar (F) object and one novel (N) object. T1 was performed at 15 min after MO (1% and 3%) inhalation. A Logitech HD Webcam C922 Pro Stream camera (Logitech, Lausanne, Switzerland) recorded animal behavior, and the videos were analyzed using ANY-maze^®^ software (Stoelting CO, Wood Dale, IL, USA). The discrimination between F and N in the T2 trial was measured by comparing the time spent in exploring F (TF) with that spent in exploring N (TN). The discrimination index (DI) represents the difference in exploration time expressed as a proportion of the total time spent exploring the two objects in T2. DI was calculated as TN – TF/TN + TF. Between trials, the objects were washed with a 10% ethanol solution. Exploration was defined as sniffing or touching the object with the nose and/or forepaws. Sitting on the object was not considered exploration.

#### 3.7.3. Radial Arm Maze

As described before by Olton and Samuelson [52] and Postu et al. [50], with the help of a radial arm maze (RAM), the spatial memory was determined for one week. The maze, comprising eight arms marked from 1 to 8 (48 cm × 12 cm), with a radial extension of 32 cm in diameter from the central area, had 50 mg food pellet at the end of arms 1, 2, 4, 5, and 7. Four days of habituation sessions were performed. Rats were instructed to move to the end of the arms and eat the food pellet during 5 min sessions. After habituation, only one trial per day was granted to all rats. For working and reference memory tasks, each rat was individually placed in the center of the maze 15 min after inhaling MO (1% and 3%). A Logitech HD Webcam C922 Pro Stream camera (Logitech, Lausanne, Switzerland) recorded animal behavior, and the videos were analyzed using ANY-maze^®^ software (Stoelting CO, Wood Dale, IL, USA). Determinations were carried out by (i) assessing the number of working memory errors (getting inside an arm that contains food pellet, but earlier stepped into) and (ii) calculating the number of reference memory errors (calculating animal entrances in an arm without a food pellet). Between trials, the radial arm maze was washed with a 10% ethanol solution.

### 3.8. Biochemical Parameters Assay

After the recording of the behavioral data, rats were euthanized, and their brains were isolated for a biochemical parameters assay. The hippocampi were precisely excised from the rat’s brains and gently homogenized in ice and 0.1 M potassium phosphate buffer (pH 7.4) with 1.15% KCl with Potter Homogenizer (Cole-Parmer, Vernon Hills, IL, USA). The homogenates were centrifuged at 960× *g* for 15 min at 4 °C. The supernatant was used for the estimation of AChE, superoxide dismutase (SOD), and catalase (CAT) specific activities, and the total content of reduced glutathione (GSH) and protein carbonyl and malondialdehyde (MDA) levels following the methods described in detail by Postu et al. [50]. Estimation of protein content was done through a bicinchoninic acid (BCA) protein assay kit (Sigma-Aldrich, Darmstadt, Germany) [53].

### 3.9. Statistical Analyses

All data are expressed as the mean ± standard error of the mean (SEM). GraphPad Prism 8.0 (GraphPad Software, Inc., San Diego, CA, USA) was used to perform statistical analyses through a one-way analysis of variance (ANOVA) followed by Tukey’s post hoc multiple comparison test, considering treatment as a factor. A *p*-value of <0.05 was considered significant. Correlation among enzymatic activities and lipid peroxidation was estimated by the Pearson correlation coefficient (*r*).

## 4. Conclusions

By using GC–MS analysis, we showed that limonene (91.11%) was the major compound within the chemical composition of the MO. The computational method has been successfully used to predict the molecular interactions of limonene with the active site of BChE and to better understand the molecular basis of the biological responses. Molecular docking simulations provided additional insights into the possible mechanism of action of limonene against BChE, which was modulated by vdW contacts between limonene and the active site residues SER198, HIS438, LEU286, VAL288, and PHE329. Furthermore, the present study demonstrated that MO was able to revert the cognitive deficits resulting from the Sco treatment. Additionally, we also established that the positive effects of MO could be mediated by decreasing brain oxidative stress and regulation of AChE and BChE activities. Therefore, the present work suggested that our MO can be regarded as an alternative source of bioactive compounds which can improve dementia-related conditions.

## References

[1] Tan E.K., Srivastava A.K., Arnold W.D., Singh M.P., Zhang Y. (2015). Neurodegeneration: Etiologies and new therapies. Biomed. Res. Int..

[2] Esquerda-Canals G., Montoliu-Gaya L., Güell-Bosch J., Villegas S. (2017). Mouse models of Alzheimer’s disease. J. Alzheimer’s Dis..

[3] A Shah A., A Dar T., A Dar P., A Ganie S., A Kamal M. (2017). A Current Perspective on the Inhibition of Cholinesterase by Natural and Synthetic Inhibitors. Curr. Drug Metab..

[4] Haake A., Nguyen K., Friedman L., Chakkamparambil B., Grossberg G.T. (2020). An update on the utility and safety of cholinesterase inhibitors for the treatment of Alzheimer’s disease. Expert Opin. Drug Saf..

[5] Tonga J.B., Eilertsen D.-E., Solem I.K.L., Arnevik E.A., Korsnes M.S., Ulstein I.D. (2020). Effect of self-efficacy on quality of life in people with Mild Cognitive Impairment and Mild Dementia: The mediating roles of depression and anxiety. Am. J. Alzheimer’s Dis. Other Dement..

[6] Cassidy L., Fernandez F., Johnson J.B., Naiker M., Owoola A.G., Broszczak D.A. (2020). Oxidative stress in alzheimer’s disease: A review on emergent natural polyphenolic therapeutics. Complement. Ther. Med..

[7] Ayaz M., Sadiq A., Junaid M., Ullah F., Subhan F., Ahmed J. (2017). Neuroprotective and Anti-Aging Potentials of Essential Oils from Aromatic and Medicinal Plants. Front. Aging Neurosci..

[8] Tongnuanchan P., Benjakul S. (2014). Essential Oils: Extraction, Bioactivities, and Their Uses for Food Preservation. J. Food Sci..

[9] Benny A., Thomas J. (2019). Essential Oils as Treatment Strategy for Alzheimer’s Disease: Current and Future Perspectives. Planta Med..

[10] De Araújo J.S.F., de Souza E.L., Oliveira J.R., Gomes A.C.A., Kotzebue L.R.V., da Silva Agostini D.L., de Oliveira D.L.V., Mazzetto S.E., da Silva A.L., Cavalcanti M.T. (2020). Microencapsulation of sweet orange essential oil (Citrus aurantium var. dulcis) by liophylization using maltodextrin and maltodextrin/gelatin mixtures: Preparation, characterization, antimicrobial and antioxidant activities. Int. J. Biol. Macromol..

[11] Falls N., Singh D., Anwar F., Verma A., Kumar V. (2018). Amelioration of neurodegeneration and cognitive impairment by lemon oil in experimental model of stressed mice. Biomed. Pharmacother..

[12] Oskouie A.A., Yekta R.F., Tavirani M.R., Kashani M.S., Goshadrou F. (2018). Lavandula angustifolia effects on rat models of Alzheimer’s disease through the investigation of serum metabolic features using NMR metabolomics. Avicenna J. Med. Biotechnol..

[13] Kennedy D., Scholey A. (2006). The psychopharmacology of European herbs with cognition-enhancing properties. Curr. Pharm. Des..

[14] Hasanein P., Felehgari Z., Emamjomeh A. (2016). Preventive effects of Salvia officinalis L. against learning and memory deficit induced by diabetes in rats: Possible hypoglycaemic and antioxidant mechanisms. Neurosci. Lett..

[15] Zhou W., Fukumoto S., Yokogoshi H. (2009). Components of lemon essential oil attenuate dementia induced by scopolamine. Nutr. Neurosci..

[16] Kim K., Bu Y., Jeong S., Lim J., Kwon Y., Cha D.S., Kim J., Jeon S., Eun J., Jeon H. (2006). Memory-enhancing effect of a supercritical carbon dioxide fluid extract of the needles of Abies koreana on scopolamine-induced amnesia in mice. Biosci. Biotechnol. Biochem..

[17] Lee G.-Y., Lee C., Park G.H., Jang J.-H. (2017). Amelioration of Scopolamine-Induced Learning and Memory Impairment by α-Pinene in C57BL/6 Mice. Evid. Based. Complement. Alternat. Med..

[18] Mukherjee P.K., Kumar V., Mal M., Houghton P.J. (2007). In vitro acetylcholinesterase inhibitory activity of the essential oil from Acorus calamus and its main constituents. Planta Med..

[19] De Souza A., Lopes E.M.C., da Silva M.C., Cordeiro I., Young M.C.M., Sobral M.E.G., Moreno P.R.H. (2010). Chemical composition and acetylcholinesterase inhibitory activity of essential oils of Myrceugenia myrcioides(Cambess.) O. Berg and Eugenia riedelianaO. Berg, Myrtaceae. Rev. Bras. Farmacogn..

[20] Dohi S., Terasaki M., Makino M. (2009). Acetylcholinesterase inhibitory activity and chemical composition of commercial essential oils. J. Agric. Food Chem..

[21] Jewart R.D., Green J., Lu C., Cellar J., Tune L.E. (2005). Cognitive, Behavioral, and Physiological Changes in Alzheimer Disease Patients as a Function of Incontinence Medications. Am. J. Geriatr. Psychiatry.

[22] Potasiewicz A., Krawczyk M., Gzielo K., Popik P., Nikiforuk A. (2020). Positive allosteric modulators of alpha 7 nicotinic acetylcholine receptors enhance procognitive effects of conventional anti-Alzheimer drugs in scopolamine-treated rats. Behav. Brain Res..

[23] Lee H.J., Lee S.K., Lee D.R., Choi B.K., Le B., Yang S.H. (2019). Ameliorating effect of Citrus aurantium extracts and nobiletin on β-amyloid (1-42)-induced memory impairment in mice. Mol. Med. Rep..

[24] Xu P., Wang K., Lu C., Dong L., Gao L., Yan M., Aibai S., Liu X. (2016). Protective effect of lavender oil on scopolamine induced cognitive deficits in mice and H2O2 induced cytotoxicity in PC12 cells. J. Ethnopharmacol..

[25] Ozarowski M., Mikolajczak P.L., Bogacz A., Gryszczynska A., Kujawska M., Jodynis-Liebert J., Piasecka A., Napieczynska H., Szulc M., Kujawski R. (2013). Rosmarinus officinalis L. leaf extract improves memory impairment and affects acetylcholinesterase and butyrylcholinesterase activities in rat brain. Fitoterapia.

[26] Lopresti A.L. (2017). Salvia (Sage): A Review of its Potential Cognitive-Enhancing and Protective Effects. Drugs R D.

[27] Kennedy D.O., Dodd F.L., Robertson B.C., Okello E.J., Reay J.L., Scholey A.B., Haskell C.F. (2011). Monoterpenoid extract of sage (Salvia lavandulaefolia) with cholinesterase inhibiting properties improves cognitive performance and mood in healthy adults. J. Psychopharmacol..

[28] Scholey A.B., Tildesley N.T.J., Ballard C.G., Wesnes K.A., Tasker A., Perry E.K., Kennedy D.O. (2008). An extract of Salvia (sage) with anticholinesterase properties improves memory and attention in healthy older volunteers. Psychopharmacol. (Berl).

[29] Bai P., Wang K., Zhang P., Shi J., Cheng X., Zhang Q., Zheng C., Cheng Y., Yang J., Lu X. (2019). Development of chalcone-O-alkylamine derivatives as multifunctional agents against Alzheimer’s disease. Eur. J. Med. Chem..

[30] Kamat P.K., Kalani A., Rai S., Swarnkar S., Tota S., Nath C., Tyagi N. (2016). Mechanism of Oxidative Stress and Synapse Dysfunction in the Pathogenesis of Alzheimer’s Disease: Understanding the Therapeutics Strategies. Mol. Neurobiol..

[31] Swomley A.M., Butterfield D.A. (2015). Oxidative stress in Alzheimer disease and mild cognitive impairment: Evidence from human data provided by redox proteomics. Arch. Toxicol..

[32] Poprac P., Jomova K., Simunkova M., Kollar V., Rhodes C.J., Valko M. (2017). Targeting Free Radicals in Oxidative Stress-Related Human Diseases. Trends Pharmacol. Sci..

[33] Praticò D. (2008). Oxidative stress hypothesis in Alzheimer’s disease: A reappraisal. Trends Pharmacol. Sci..

[34] Haider S., Liaquat L., Ahmad S., Batool Z., Siddiqui R.A., Tabassum S., Shahzad S., Rafiq S., Naz N. (2020). Naringenin protects AlCl3/D-galactose induced neurotoxicity in rat model of AD via attenuation of acetylcholinesterase levels and inhibition of oxidative stress. PLoS ONE.

[35] Youn K., Lee S., Jun M. (2019). Discovery of Nobiletin from Citrus Peel as a Potent Inhibitor of β-Amyloid Peptide Toxicity. Nutrients.

[36] Mushtaq A., Anwar R., Ahmad M. (2018). Lavandula stoechas (L) a very potent antioxidant attenuates dementia in scopolamine induced memory deficit mice. Front. Pharmacol..

[37] Ding Y., Zhang Z., Yue Z., Ding L., Zhou Y., Huang Z., Huang H. (2019). Rosmarinic acid ameliorates H2O2-induced oxidative stress in L02 cells through MAPK and Nrf2 pathways. Rejuvenation Res..

[38] Capatina L., Boiangiu R.S., Dumitru G., Napoli E.M., Ruberto G., Hritcu L., Todirascu-Ciornea E. (2020). Rosmarinus officinalis Essential Oil Improves Scopolamine-Induced Neurobehavioral Changes via Restoration of Cholinergic Function and Brain Antioxidant Status in Zebrafish (Danio rerio). Antioxidants.

[39] Kolac U.K., Ustuner M.C., Tekin N., Ustuner D., Colak E., Entok E. (2017). The anti-inflammatory and antioxidant effects of Salvia officinalis on lipopolysaccharide-induced inflammation in rats. J. Med. Food.

[40] Bahri S., Ben Ali R., Nahdi A., Mlika M., Abdennabi R., Jameleddine S. (2019). Salvia officinalis attenuates bleomycin-induced oxidative stress and lung fibrosis in rats. Nutr. Cancer.

[41] Wang Y., Qian J., Cao J., Wang D., Liu C., Yang R., Li X., Sun C. (2017). Antioxidant capacity, anticancer ability and flavonoids composition of 35 citrus (Citrus reticulata Blanco) varieties. Molecules.

[42] Xu P., Wang K., Lu C., Dong L., Gao L., Yan M., Aibai S., Yang Y., Liu X. (2017). The Protective Effect of Lavender Essential Oil and Its Main Component Linalool against the Cognitive Deficits Induced by D-Galactose and Aluminum Trichloride in Mice. Evid. -Based Complement. Altern. Med..

[43] Habtemariam S. (2016). The therapeutic potential of rosemary (Rosmarinus officinalis) diterpenes for Alzheimer’s disease. Evidence-Based Complement. Altern. Med..

[44] Ghorbani A., Esmaeilizadeh M. (2017). Pharmacological properties of Salvia officinalis and its components. J. Tradit. Complement. Med..

[45] Brus B., Košak U., Turk S., Pišlar A., Coquelle N., Kos J., Stojan J., Colletier J.-P., Gobec S. (2014). Discovery, Biological Evaluation, and Crystal Structure of a Novel Nanomolar Selective Butyrylcholinesterase Inhibitor. J. Med. Chem..

[46] Friesner R.A., Murphy R.B., Repasky M.P., Frye L.L., Greenwood J.R., Halgren T.A., Sanschagrin P.C., Mainz D.T. (2006). Extra Precision Glide: Docking and Scoring Incorporating a Model of Hydrophobic Enclosure for Protein−Ligand Complexes. J. Med. Chem..

[47] Ellman G., Courtney K., Andres V.J., Feather-Stone R. (1961). A new and rapid colorimetric determination of acetylcholinesterase activity. Biochem. Pharm..

[48] Ionita R., Postu P.A., Mihasan M., Gorgan D.L., Hancianu M., Cioanca O., Hritcu L. (2018). Ameliorative effects of Matricaria chamomilla L. hydroalcoholic extract on scopolamine-induced memory impairment in rats: A behavioral and molecular study. Phytomedicine.

[49] Jackson L.L. (1943). VTE on an elevated T-maze. J. Comp. Psychol..

[50] Postu P.A., Sadiki F.Z., El Idrissi M., Cioanca O., Trifan A., Hancianu M., Hritcu L. (2019). Pinus halepensis essential oil attenuates the toxic Alzheimer’s amyloid beta (1-42)-induced memory impairment and oxidative stress in the rat hippocampus. Biomed. Pharmacother..

[51] Foyet H.S., Asongalem A.E., Oben E.K., Cioanca O., Hancianu M., Hritcu L. (2016). Effects of the Methanolic Extract of Vitellaria paradoxa Stem Bark Against Scopolamine-Induced Cognitive Dysfunction and Oxidative Stress in the Rat Hippocampus. Cell. Mol. Neurobiol..

[52] Olton D.S., Samuelson R.J. (1976). Remembrance of places passed: Spatial memory in rats. J. Exp. Psychol. Anim. B.

[53] Smith P.K., Krohn R.I., Hermanson G.T., Mallia A.K., Gartner F.H., Provenzano M.D., Fujimoto E.K., Goeke N.M., Olson B.J., Klenk D.C. (1985). Measurement of protein using bicinchoninic acid. Anal. Biochem..

